# Intricate effects of post-translational modifications in liver cancer: mechanisms to clinical applications

**DOI:** 10.1186/s12967-024-05455-6

**Published:** 2024-07-13

**Authors:** Yu Zhang, Weihao Xu, Chuanhui Peng, Shenli Ren, Cheng Zhang

**Affiliations:** Division of Hepatobiliary and Pancreatic Surgery, Department of Surgery, The First Affiliated Hospital, Zhejiang University School of Medicine, Hangzhou, Zhejiang China

**Keywords:** Hepatocellular carcinoma, Post-translational modifications, Expression changes, Pathogenic mechanisms, Clinical applications

## Abstract

Liver cancer is a significant global health challenge, with hepatocellular carcinoma (HCC) being the most prevalent form, characterized by high incidence and mortality rates. Despite advances in targeted therapies and immunotherapies, the prognosis for advanced liver cancer remains poor. This underscores the urgent need for a deeper understanding of the molecular mechanisms underlying HCC to enable early detection and the development of novel therapeutic strategies. Post-translational modifications (PTMs) are crucial regulatory mechanisms in cellular biology, affecting protein functionality, interactions, and localization. These modifications, including phosphorylation, acetylation, methylation, ubiquitination, and glycosylation, occur after protein synthesis and play vital roles in various cellular processes. Recent advances in proteomics and molecular biology have highlighted the complex networks of PTMs, emphasizing their critical role in maintaining cellular homeostasis and disease pathogenesis. Dysregulation of PTMs has been associated with several malignant cellular processes in HCC, such as altered cell proliferation, migration, immune evasion, and metabolic reprogramming, contributing to tumor growth and metastasis. This review aims to provide a comprehensive understanding of the pathological mechanisms and clinical implications of various PTMs in liver cancer. By exploring the multifaceted interactions of PTMs and their impact on liver cancer progression, we highlight the potential of PTMs as biomarkers and therapeutic targets. The significance of this review lies in its potential to inform the development of novel therapeutic approaches and improve prognostic tools for early intervention in the fight against liver cancer.

## Introduction

Liver cancer is the sixth most prevalent cancer (4.3% of all cancer sites) and the third leading cause of cancer-related mortality (7.8% of all cancer sites) worldwide, according to Global Cancer Statistics 2022 [1–3]. Hepatocellular carcinoma (HCC) accounts for 75–85% of liver cancer cases. The incidence of liver cancer shows notable geographical variations, with East Asia and Sub-Saharan Africa experiencing the highest rates, largely due to the varying prevalence of risk factors such as hepatitis B virus (HBV) and hepatitis C virus (HCV) infections, aflatoxin exposure, chronic alcohol use, and non-alcoholic fatty liver disease (NAFLD) [4–7]. Recent strides in molecular biology have shed light on the complex interplay of genetic, epigenetic, and environmental factors in liver cancer pathogenesis [8–11]. The classification of HCC into molecular subtypes based on genetic variations has introduced precision medicine, enabling treatment customization according to the unique genetic features of individual patients [12–15]. The emergence of immunotherapy, particularly checkpoint inhibitors like nivolumab and pembrolizumab, has marked a new era in HCC treatment, offering hope for better outcomes [12, 16–19]. However, challenges such as tumor heterogeneity, late-stage diagnosis, and resistance to systemic therapies persist, contributing to an overall five-year survival rate of less than 20% for patients with liver cancer [9, 20, 21]. These challenges underscore the urgent need for innovative diagnostic tools and therapeutic strategies, emphasizing the critical role of ongoing research in unraveling the mechanisms of liver cancer.

**Fig. 1:**
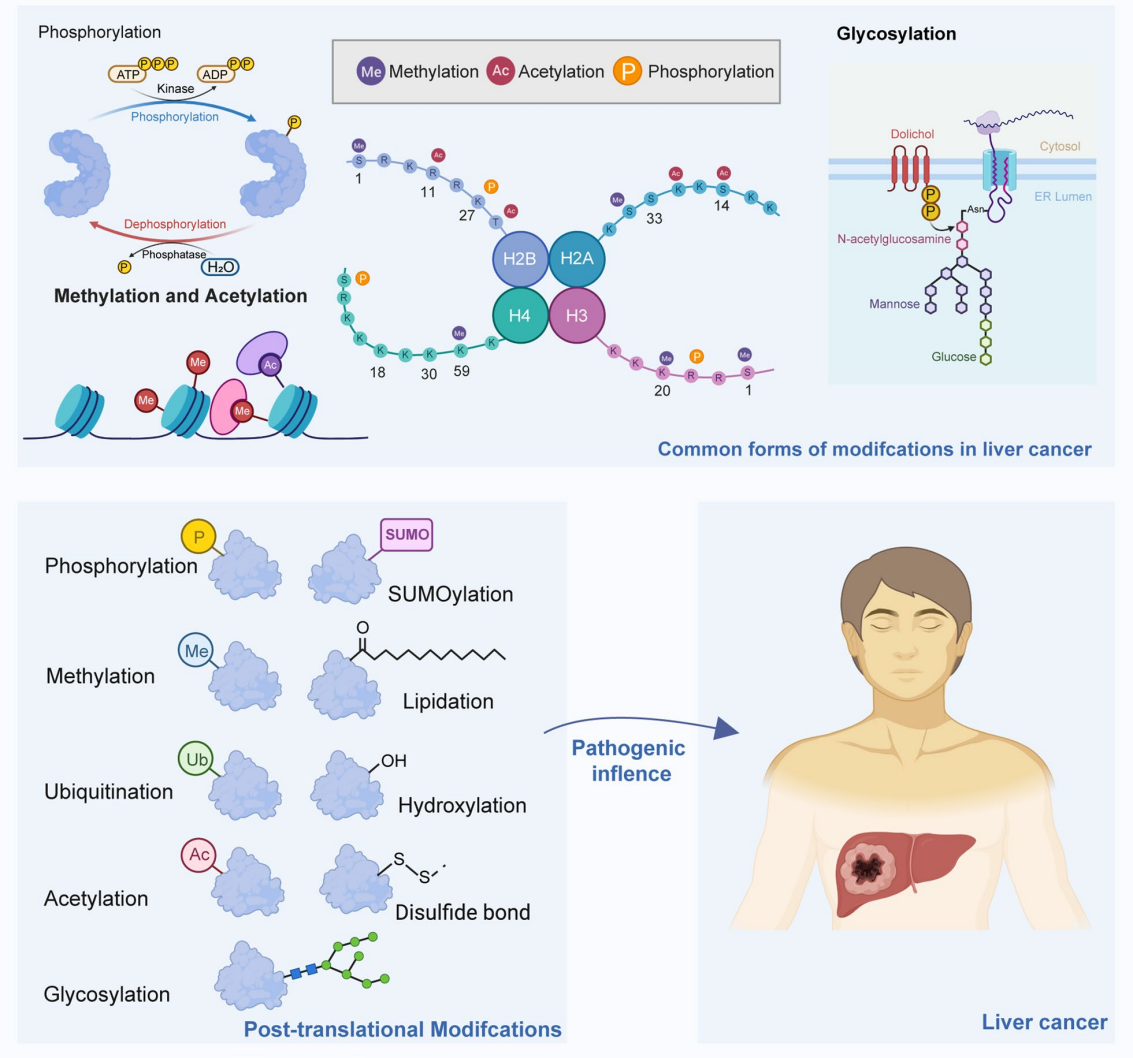
Landscape of post-translational modifications in liver cancer. Various post-translational modifications (PTMs) are instrumental in the progression of liver cancer, modulating the function and activity of target molecules involved in a range of malignant biological processes. These processes encompass but are not limited to cell proliferation, migration and invasion, autophagy, metabolic reprogramming, chemoresistance, and immune evasion. The diverse regulatory mechanisms of PTMs in liver cancer highlight their critical roles in tumor progression. Insights into the epigenetic landscape obtained by exploring PTMs in liver cancer have significant implications for developing targeted therapies, prognostic assessment, drug efficacy prediction, and early clinical diagnosis

Post-translational modifications (PTMs), the covalent addition of specific chemical groups to proteins after translation, represent a sophisticated mechanism for controlling protein function, localization, and interactions [22–25]. These modifications, which include phosphorylation, acetylation, ubiquitination, methylation, and glycosylation, dynamically alter protein activities, enabling cells to precisely respond to various stimuli [24, 26–28]. Among these, phosphorylation, which is critical for cellular processes, is the most extensively studied PTM [29–33]. The balance between kinases and phosphatases is vital for maintaining cellular signaling integrity. Meanwhile, acetylation affects gene expression by modifying chromatin accessibility, influencing several biological processes [34–37]. Methylation predominantly occurs within the cell nucleus and on nuclear proteins, where lysine and arginine residues are the principal targets [38–40]. Glycosylation involves the enzymatic attachment of sugar moieties to serine and/or threonine residues on proteins. It encompasses three prevalent forms: N-glycosylation, O-glycosylation, and glypiation [41–45]. Advancements in proteomics have deepened the understanding of PTMs, revealing their extensive involvement in both physiological processes and disease progression, including cancer [46–48]. Emerging research has highlighted that PTMs contribute to HCC tumorigenesis and cancer progression by influencing cellular proliferation, apoptosis, invasion, deoxyribonucleic acid (DNA) repair, autophagy, metabolism, chemotherapy resistance, and immune evasion (Fig. 1) [49–52]. For example, dysregulation of phosphorylation controls critical signaling pathways involved in cell growth and apoptosis [53, 54]. Acetylation affects chromatin structure and gene expression, whereas ubiquitination regulates protein degradation, influencing cell cycle progression and DNA damage response [37, 55]. Methylation has emerged as a key player in modulating the complex interplay between genetic and environmental factors that drive tumor initiation, progression, and resistance to therapies [38, 56, 57]. Glycosylation affects cell-to-cell and cell-to-matrix interactions, crucial for tumor metastasis (Table 1) [58–60]. Inhibiting specific enzymes responsible for the addition or removal of PTMs has unveiled the complex interplay between PTMs and liver cancer and revealed promising avenues for advancements in the diagnosis, prognostic prediction, and targeted therapies of liver cancer [48, 61].

**Table 1:** Effects and regulatory mechanisms of post-translational modifications in the carcinogenesis of liver cancer

Modified type	Expression change	Regulator	Protein	Related mechanisms	Role	Functions	Publication year	Reference
Phosphorylation	Upregulated	CKB T133	GPX4	IGF1R/AKT/CKB/GPX4	Carcinogenic	Promote cell viability and tumor growth	2023	[68]
Phosphorylation	Upregulated	AK2	LOX3	EGF-EGFR/TOM20/AK2/LOX3/DHODH	Carcinogenic	Promote cell viability and chemoresistance to oxaliplatin	2023	[69]
Phosphorylation	Upregulated	Metformin	DOCK1	Metformin/DOCK1/RAC1	Carcinogenic	Promote cell viability and resistance to metformin	2022	[71]
Phosphorylation	Upregulated	PCK1	INSIG1/2	AKT/PCK1/INSIG1/2/SREBP1	Carcinogenic	Promote cell proliferation, lipogenesis, and tumorigenesis	2020	[70]
Phosphorylation	Upregulated	PKCα	ZFP64	PKCα/ZFP64/CSF1	Carcinogenic	Promote macrophages to the M2 phenotype, immune escape, and anti-PD1 tolerance	2022	[72]
Acetylation	Upregulated	Sirtuin 2	FGL1	Sirtuin 2/FGL1	Carcinogenic	Promote immune evasion, tumor growth, and overall survival	2023	[78]
Acetylation	Upregulated	PCAF	PGK1	PCAF/PGK1	Carcinogenic	Promote cell glycolysis, proliferation, and tumorigenesis	2017	[79]
Acetylation	Downregulated	GCN5L1	GLS1, and GLS2	GCN5L1/GLS1/GLS2/mTORC1	Tumor suppressor	Inhibit glutaminolysis, cell proliferation, and tumor growth	2022	[80]
Acetylation	Upregulated	SCARB2	MYC	SCARB2/MYC	Carcinogenic	Promote cell proliferation, invasion, stem cell-like characteristics, and tumorigenesis	2023	[89]
Acetylation	Upregulated	IL‐6	GαS	IL‐6/GαS/STAT3	Carcinogenic	Drive hepatocarcinogenesis	2023	[81]
Methylation	Upregulated	PRMT9	HSPA8	HBx/PRMT9/HSPA8/CD44	Carcinogenic	Inhibit ferroptosis, promote cell proliferation and tumor growth	2023	[96]
Methylation	Upregulated	PRMT3	IGF2BP1	PRMT3/IGF2BP1/HEG1	Carcinogenic	Promote cell proliferation and chemoresistance to oxaliplatin	2023	[93]
Methylation	Upregulated	PRMT1	PHGDH	PRMT1/PHGDH/serine	Carcinogenic	Promote serine synthesis, cell proliferation, and tumor growth	2023	[95]
Methylation	Upregulated	PRMT5	RORα	ROS/PRMT5/ITCH/RORα	Carcinogenic	Promote cell proliferation, cell migration, and invasion	2023	[110]
Methylation	Upregulated	PRMT1	PFKFB3	TK1/TRIM48/PRMT1/PFKFB3	Carcinogenic	Promote glycolysis, cell proliferation, tumor growth, and metastasis	2023	[94]
Glycosylation	Upregulated	B3GALT5	mTOR	B3GALT5/mTOR/p70s6k	Carcinogenic	Promote glycolysis, cell proliferation, and tumor growth	2022	[112]
Glycosylation	Upregulated	GALNT1	MMP14	GALNT1/MMP14	Carcinogenic	Promote tissue invasion, metastases, and ECM degradation	2017	[114]
Glycosylation	Upregulated	SLC35A2	B4GalT1	SLC35A2/B4GalT1	Carcinogenic	Promote cell invasion, and metastasis	2023	[113]
Glycosylation	Downregulated	O-GlcNAcylation	PARG	PARG O-GlcNAcylation/DDB1/c-Myc	Tumor suppressor	Inhibit tumor growth	2023	[115]
Glycosylation	Downregulated	ST6GAL1	MCAM	ST6GAL1/MCAM	Tumor suppressor	Inhibit cell migration, invasion, and tumor metastasis	2023	[118]

This review aims to highlight the critical role of PTMs in the pathogenesis and progression of liver cancer, emphasizing the most extensively explored PTMs, mainly including phosphorylation, acetylation, methylation, and glycosylation. By dissecting the intricate mechanisms through which PTMs influence key cellular processes in liver cancer, the paper seeks to elucidate their potential as therapeutic targets, discuss the challenges in targeting these modifications, and explore promising research directions (Table 2). Ultimately, this review aims to improve diagnostic accuracy, prognostic prediction, and the personalization of treatment strategies for liver cancer by leveraging the insights gained from the study of PTMs in HCC pathogenesis.

**Table 2:** Clinical significance of post-translational modifications in liver cancer

Modified type	Regulator	Target	Role	Samples	Clinical application(s)	Clinical relevance	Publication year	Reference
Phosphorylation	CKB T133	GPX4	Carcinogenic	293 T, THLE-2, MDA-MB-231, A549, HCT116, LN18, and LN229 cells, human HCC and adjacent matched non-tumor tissues, xenograft mouse models, and orthotopic mouse models	Predict the prognosis	Unfavorable prognosis (overall survival)	2023	[68]
Phosphorylation	AK2	LOX3	Carcinogenic	Huh7 and Hep3B cells, S704D-LOXL3 mice, human cancer and adjacent tissues of 50 patients with HCC	Predict the prognosis	High-grade pathological grading and worse prognosis	2023	[69]
Phosphorylation	PCK1	INSIG1/2	Carcinogenic	Hep3B, Huh7, H1993, CHL-1, SNU-398, SNU-475, HL7702, THLE-2, and 293 T cells, human HCC and adjacent matched non-tumor tissues, xenograft mouse models, and orthotopic mouse models	Predict the prognosis	Unfavorable prognosis (overall survival)	2020	[70]
Phosphorylation	PKCα	ZFP64	Carcinogenic	Human HCC HCCLM3, MHCC97H, MHCC97L, HepG2, PLC/PRF/5, mouse HCC Hepa1-6 cells, spontaneously tumorigenic transgenic mouse models, and human HCC puncture specimens	Predict the prognosis and anti-PD1 therapy	Anti-PD1 tolerance and unfavorable prognosis (overall survival and disease-free survival)	2022	[72]
Acetylation	Sirtuin 2	FGL1	Carcinogenic	HEK293T, HCCLM3, SMMC-7721, Huh7, Hepa 1–6, and H22 cells, Hepa 1–6 and H22 cancer mouse models, and human HCC tissues and paired adjacent tissues	Predict the prognosis; anti-PD1 therapy	Enhance HCC immunotherapy and unfavorable prognosis (tumor stage, size, and overall survival)	2023	[78]
Acetylation	PCAF	PGK1	Carcinogenic	HepG2, HCCLM3, Huh7, HL7702, SNU739, SNU182, JHH5, JHH4, Huh28, SNU449, SNU421, SNU886, SNU761, and SNU739 cells, mouse xenograft models, and human HCC tissues and paired adjacent tissues	Predict the prognosis	unfavorable prognosis (overall survival and recurrence)	2017	[79]
Acetylation	GCN5L1	GLS1 and GLS2	Tumor suppressor	Murine hepa1‐6 and hepa1c1c7 cells, human HepG2 and Huh7 cells, DEN and CCl4‐induced HCC mouse models, and human HCC tissues and paired adjacent normal tissues	Predict the prognosis	Unfavorable prognosis (overall survival, progression‐free survival, and disease‐free survival)	2022	[80]
Acetylation	IL‐6	GαS	Carcinogenic	hcPCs, HCC mouse models, and human HCC tissues and paired adjacent normal tissues	Predict the prognosis and prevent hepatocarcinogenesis	Unfavorable prognosis (tumor stage, histological grade, overall survival, and disease‐free survival)	2023	[81]
Methylation	PRMT9	HSPA8	Carcinogenic	HepG2, MHCC97H, and Huh7 cells, murine HCC models, and human HCC tumor samples and adjacent normal tissues	Predict the prognosis	Unfavorable prognosis (overall survival)	2023	[96]
Methylation	PRMT3	IGF2BP1	Carcinogenic	PLC-8024, Huh7, and HepG2 cells, murine HCC models, patient-derived cell (PDC) models, and human HCC tissues	Predict the prognosis; oxaliplatin resistance	Unfavorable prognosis (tumor stage, histological grade, overall survival, and disease‐free survival)	2023	[93]
Methylation	PRMT1	PHGDH	Carcinogenic	Huh7, PLC/PRF/5, and HEK293T cells, murine HCC models, and human HCC tissues	Predict the prognosis; therapeutic target	TAT-tagged nonmethylated peptide and unfavorable prognosis (overall survival)	2023	[95]
Methylation	PRMT1	PFKFB3	Carcinogenic	Murine HEK 293 T cells, human HCC cell lines (MHCC97H, Hep3B, MHCC97L, HCCLM3, and Huh7), murine HCC models, and human HCC tissues and adjacent paired non-tumor tissues	Predict the prognosis; therapeutic target	Unfavorable prognosis (tumor volume, microvascular invasion, TNM stage, overall survival, and disease‐free survival)	2023	[94]
Glycosylation	B3GALT5	mTOR	Carcinogenic	HUH7, PLC/PRF5, HCCLM3, and HLE cells, murine HCC models, and human HCC tissues and adjacent paired non-tumor tissues	Predict the prognosis; therapeutic target	Unfavorable prognosis (overall survival)	2022	[112]
Glycosylation	GALNT1	MMP14	Carcinogenic	HepG2 cells, murine HCC models, and human HCC tissues and adjacent paired non-tumor tissues	Predict the prognosis	Unfavorable prognosis (overall survival)	2017	[114]
Glycosylation	SLC35A2	B4GalT1	Carcinogenic	Hep3B, HepG2, MHCC97L, MHCC97H, and HCCLM6 cells and human HCC tissues and adjacent paired non-tumor tissues	Predict the prognosis	Unfavorable prognosis (overall survival)	2023	[113]
Phosphorylation	Metformin	DOCK1	Carcinogenic	Liver cancer cell lines (HEK293T, PLC, Hep3B, Huh7, SNU423, SNU449, and SNU475), human HCC and adjacent nontumor liver tissues, patient-derived HCC organoids, xenograft mouse models, and orthotopic mouse models	Personalized therapeutic strategy	/	2022	[71]
Acetylation	SCARB2	MYC	Carcinogenic	HepG2, Huh7, Hep3B, Hepa1-6, H22, 293FT, and HEK 293 T cells, tumor organoids, and HCC mouse models	Therapeutic target	Brefeldin A	2023	[89]
Methylation	PRMT5	RORα	Carcinogenic	HepG2 cells, and murine HCC models	Therapeutic target	/	2023	[110]
Glycosylation	O-GlcNAcylation	PARG	Tumor suppressor	U2OS and 293 T cells, and murine HCC models	/	/	2023	[115]
Glycosylation	ST6GAL1	MCAM	Tumor suppressor	Hep3B, Huh7, SMMC-7721, YY-8103, HepG2, MHCC97L, MHCC97H, 293 T, and HCCLM3 cells and human HCC tissues and adjacent paired non-tumor tissues	/	/	2023	[118]

## Literature search strategy

To identify relevant and high-quality literature, we performed an extensive search across databases including PubMed, Scopus, and Web of Science. Our search was confined to articles published from January 2017 to April 2024. We used keywords such as “liver cancer,” “hepatocellular carcinoma,” “post-translational modifications,” “phosphorylation,” “acetylation,” “methylation,” “ubiquitination,” and “glycosylation.” We selected articles based on their pertinence to PTMs in HCC, emphasizing those that offer insights into molecular mechanisms, diagnostic implications, and therapeutic potential. After conducting the keyword searches, we focused on reviewing high-impact and high-quality articles. We prioritized studies that were most relevant to our research, ensuring a comprehensive coverage of the role of PTMs in liver cancer. This thorough screening process allowed us to incorporate the most pertinent findings and provide a detailed understanding of the role of PTMs in liver cancer.

## Expression and role of PTMs in liver cancer

### Phosphorylation and its implications in liver cancer

Increasing research has revealed a significant correlation between PTMs and the clinicopathological features and prognosis of patients with liver cancer [61–64]. Phosphorylation, as a pivotal PTM, is reported to have a strong association with tumor pathological grading and poor prognosis in liver cancer [65–69]. Among the proteins studied, creatine kinase B (CKB) shows markedly higher expression in HCC cell lines Huh7, liver cancer metastasis 3 (HCCLM3) compared to normal liver cells, suggesting its oncogenic potential [68]. Analysis of The Cancer Genome Atlas (TCGA) database and immunohistochemistry (IHC) staining of HCC samples confirmed CKB’s upregulation in tumors, correlating high CKB phosphorylation with poorer patient survival outcomes. After pathological grading of human HCC samples based on intrahepatic metastasis levels, it was observed that LOXL3 phosphorylation at S704 was markedly elevated in high-grade tissues [69]. Furthermore, increased levels of pLOXL3-Ser704 were associated with a poorer prognosis and greater resistance to chemotherapy in HCC patients. Moreover, increased phosphorylation of phosphoenolpyruvate carboxykinase 1 (PCK1) correlates with reduced survival rates of patients with HCC, positioning PCK1 phosphorylation as a potential prognostic marker for HCC outcomes [70].

**Fig. 2:**
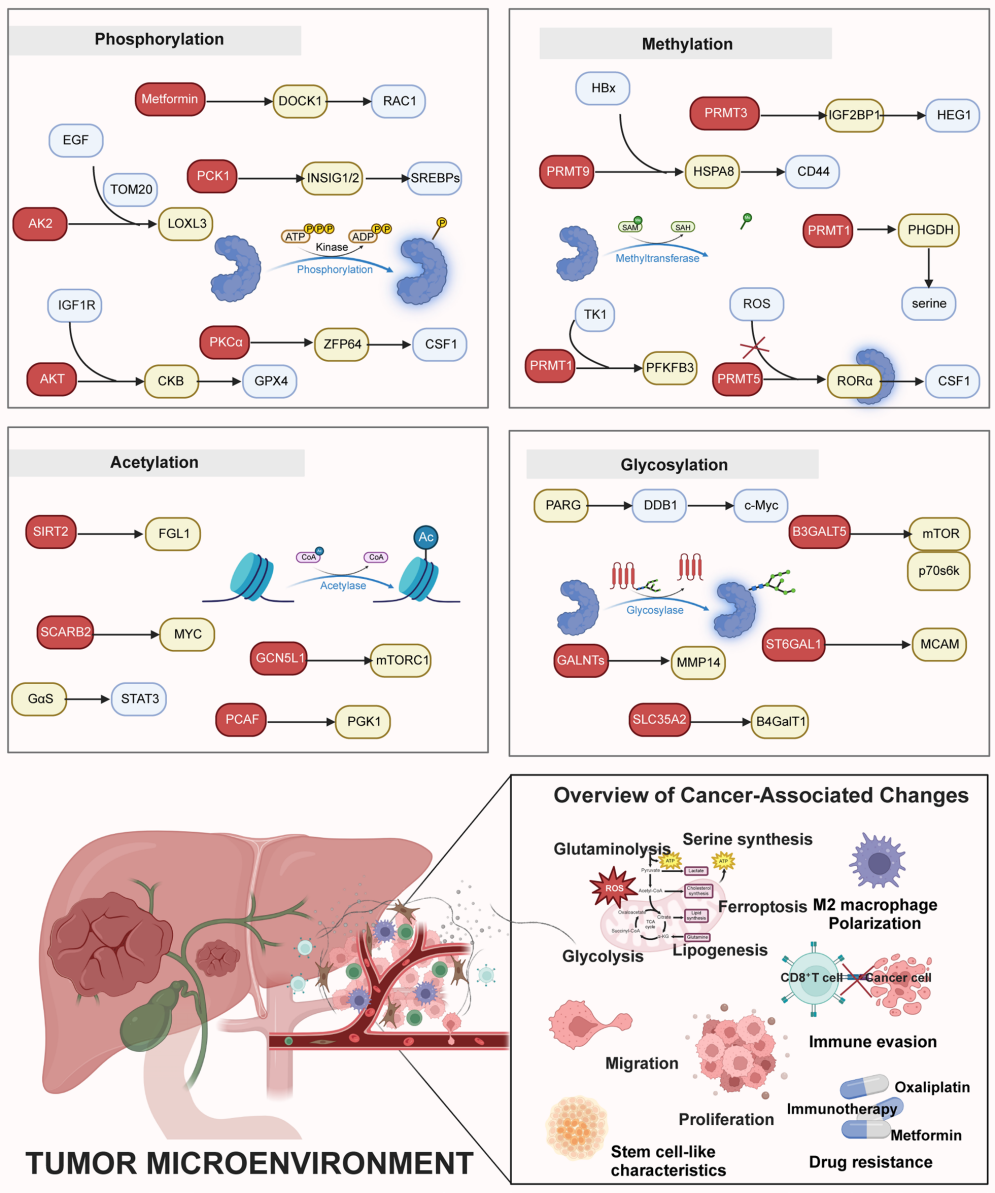
Intricate mechanisms of several common post-translational modifications in liver cancer. Phosphorylation, acetylation, ubiquitination, methylation, and glycosylation are prominently explored PTMs that significantly influence a wide array of molecular mechanisms, playing pivotal roles in the initiation and progression of liver cancer. These modifications alter protein function, stability, localization, and interactions, thereby modulating key signaling pathways and cellular processes. In liver cancer, phosphorylation activates oncogenic molecules such as GPX4, LOX3, DOCK1, INSIG1/2, and ZFP64, thereby promoting cell proliferation, survival, lipogenesis, immune escape, and resistance to oxaliplatin, metformin, and immunotherapy. Acetylation participates in modifying genes involved in cell glycolysis, proliferation, glutaminolysis, invasion, stem cell-like characteristics, and immune evasion in liver cancer. Arginine methylation, mediated by diverse PRMTs, influences various molecular pathways and cellular processes critical in cancer development, including cell proliferation, ferroptosis, serine synthesis, glycolysis, cell migration, invasion, and resistance to oxaliplatin. Alterations in glycosylation patterns modulate the activity and localization of cell surface receptors and adhesion molecules, thereby contributing to various cellular processes, including glycolysis, cell proliferation, cell invasion, adhesion, and metastasis. The intricate relationship between post-translational modifications and liver cancer progression highlights its potential as a target for therapeutic intervention

Mechanistically, recent studies have highlighted the critical role of abnormal protein phosphorylation in HCC progression, influencing cell proliferation, metabolism, immune evasion, and chemotherapy resistance [68–71]. For example, insulin-like growth factor 1 receptor (IGF1R) activation leads to protein kinase B (AKT)-mediated CKB T133 phosphorylation and glutathione peroxidase 4 (GPX4) S104 phosphorylation, enhancing cell survival by inhibiting ferroptosis and lipid peroxidation. This process underscores the importance of the AKT/CKB/GPX4 axis in developing resistance to oxidative stress in HCC [68]. Additionally, research has revealed that oxaliplatin resistance in HCC involves the phosphorylation of lysyl oxidase-like 3 (LOXL3). LOXL3, upon activation by epidermal growth factor (EGF) and its receptor (EGFR) signaling, contributes to its translocation into mitochondria through the mitochondrial import receptor translocase of outer mitochondrial membrane 20 (TOM20). The mitochondrial kinase adenylate kinase 2 (AK2) phosphorylates LOXL3 at S704, which stabilizes dihydroorotate dehydrogenase (DHODH) and enables HCC cells to resist oxaliplatin-induced ferroptosis [69]. Combining oxaliplatin with inhibitors targeting the LOXL3-DHODH axis effectively suppressed tumor growth in mouse models with advanced HCC with the LOXL3-S704D mutant, highlighting a novel resistance mechanism and therapeutic strategy. Metformin’s anti-tumor effects on various cancer types have revealed the role of dedicator of cytokinesis protein 1 (DOCK1), a canonical guanine nucleotide exchange factor (GEF) for Ras-related C3 botulinum toxin substrate (RAC) family small guanosine triphosphatases (GTPases), in influencing metformin’s efficacy in HCC [71]. Metformin-induced phosphorylation of DOCK1 at Y722 and Y1811 increases the level of RAC1-GTP to activate RAC1 in HCC PLC, SNU-449, and Hep3B cells, contributing to increased cell survival and resistance to metformin. However, combining metformin with the DOCK1 inhibitor (TBOPP) significantly reduces HCC cell viability, suggesting a synergistic approach to enhancing metformin’s anti-cancer effects. Furthermore, the combination of metformin and TBOPP has exhibited a dramatically synergistic inhibition on cell viability in both in vivo PLC, SNU449, and Hep3B cells and patient-derived HCC organoids. This introduces a promising therapeutic strategy of integrating metformin with targeted inhibition of specific signaling pathways, such as the DOCK1-mediated activation of RAC1, to potentiate the antitumor effects of metformin in liver cancer. Recent insights also connect phosphorylation with macrophage M2 polarization and anti-programmed cell death 1 (anti-PD1) resistance [72]. The studies have discovered the upregulation of zinc finger protein 64 (ZFP64) in tumor tissues of patients with HCC resistance to anti-PD1 therapy. Protein kinase C alpha (PKCα) directly phosphorylates ZFP64 at S226, facilitating its nuclear translocation and transcription activation of colony-stimulating factor 1 (CSF1). CSF1 promotes the recruitment and polarization of M2 macrophage, fostering an immunosuppressive tumor microenvironment (TME). The application of protein kinase inhibitors such as Gö6976 and lenvatinib has been shown to reset TME to favor immune-mediated tumor suppression and restore cell sensitivity to anti-PD1 therapy [15]. In Huh7 and Hep3B HCC cells, AKT prompts PCK1 phosphorylation at S90 to translocate to the endoplasmic reticulum (ER), where it respectively phosphorylates ER anchor proteins INSIG1/2 at S207 and S151. This phosphorylation diminishes sterol binding of INSIG1/2, leading to sterol regulatory element-binding proteins (SREBPs) activation and subsequent enhanced lipogenesis and tumor growth [70]. These findings not only elucidate the multifaceted role of phosphorylation in HCC but also offer potential targets for prognosis prediction and therapeutic intervention, emphasizing the need for integrated strategies to tackle HCC’s complexity (Fig. 2).

### Acetylation and its implications in liver cancer

In recent years, advancements in precision medicine have revealed abnormal acetylation levels in liver cancer, underscoring its potential prognostic value by correlating with clinicopathological factors [73–77]. For example, elevated levels of Sirtuin 2 (SIRT2) in HCC, as opposed to adjacent normal tissues, are associated with more aggressive disease features and poor prognosis, such as advanced tumor stage and larger size, highlighting their roles in HCC progression [78]. Moreover, alterations in the expression of phosphoglycerate kinase 1 (PGK1) and its acetylation by p300/CBP-associated factor (PCAF) are significantly upregulated in HCC tissues. This overexpression correlates with decreased survival rates and increased recurrence risk, underscoring its oncogenic role in liver cancer [79]. Research also shows that lower expression of the mitochondrial protein general control of amino acid synthesis 5 like 1 (GCN5L1) in HCC tissues correlates with increased glutaminase (GLS1/GLS2) acetylation and poorer patient survival, highlighting GCN5L1 as a potential prognostic indicator [80]. A significant upregulation in both the expression and acetylation of the stimulatory G protein alpha subunit (GαS protein) has been documented in dysplastic nodules and HCC tissues. Elevated levels of GαS protein are indicative of advanced tumor stages and poorer histological differentiation, inversely affecting both overall and disease-free survival outcomes. Cox proportional hazards regression analysis identifies high GαS levels as an independent prognostic factor for adverse outcomes in patients with HCC [81].

In the context of liver cancer, altered acetylation profiles have been found to influence cancer cell metabolism and the tumor microenvironment, offering insights into the metabolic vulnerabilities of liver cancer cells [74, 77, 82, 83]. It has been acknowledged that lymphocyte-activation gene 3 (LAG-3), a transmembrane protein on activated T cells, inhibits antigen-specific T cell activation by interacting with fibroblast growth factor-inducible 14 (FGL1). Research across various HCC cell lines has shown that SIRT2 enhances immune evasion by deacetylating FGL1, thus increasing its protein stability [78]. Further research demonstrates that inhibiting SIRT2 with AGK2, in conjunction with programmed death-ligand 1 (PD-L1) blockade, enhances FGL1 acetylation, restoring tumor-infiltrating cluster of differentiation 8 (CD8 +) T cell populations, suppressing tumor growth, and boosting survival in mouse models. Remarkably, aspirin has been shown to amplify the effects of PD-L1 blockade in HCC Hepa 1–6 and H22 tumor models by directly acetylating FGL1, facilitating its degradation and offering a potential strategy to enhance HCC immunotherapy [78, 84]. The increased acetylation of PGK1 at K323 has been identified as a critical oncogenic mechanism, enhancing its role in cancer progression. Importantly, the depletion of PGK1 markedly reduces cancer glycolysis, cell proliferation, and tumorigenesis of HCC in mouse xenograft models [79]. This evidence points to the potential of targeting PGK1 and its post-translational modifications as a novel therapeutic approach in liver cancer treatment. Additionally, glutamine addiction is a key metabolic pathway promoting cancer cell proliferation in HCC. In HCC models induced by the combination of diethylnitrosamine (DEN) and carbon tetrachloride (CCL4), mitochondrial GCN5L1 inhibits tumor growth by reducing GLS1/2 acetylation and activity, highlighting its role in regulating glutamine addiction during HCC progression. The loss of GCN5L1 enhances glutaminolysis and activates the mechanistic target of rapamycin C1 (mTORC1) pathway, fueling cell proliferation and tumor development [80]. Targeting GCN5L1 to disrupt HCC’s metabolic reliance on glutamine offers a promising therapeutic strategy by exploiting metabolic vulnerabilities in liver cancer. Increasing studies underscore the involvement of cancer stem cells (CSCs) in multiple malignant processes during HCC development, including tumor initiation, relapse, metastasis, and drug resistance [85–88]. Recent studies have highlighted the pivotal role of scavenger receptor class B member 2 (SCARB2) in maintaining the stem-like characteristics of HCC cells. The interaction between SCARB2 and MYC facilitates the acetylation of MYC and enhances its transcriptional activity, leading to increased proliferation of HCC cells, the formation of colonies, and the preservation of cancer stem cell-like properties. Deleting SCARB2 significantly hampers tumor growth and metastasis, driven by oncogenic MYC activation. Intervening in the SCARB2-MYC pathway through brefeldin A administration showcases a potent targeted therapy for liver cancer by targeting stem cell-like properties [89]. Furthermore, acetylation modifications have also been linked to the malignant transformation of hepatocellular carcinoma progenitor cells (hcPCs) into established HCC. Elevated levels of GαS protein in dysplastic nodules and HCC tissues, demonstrating a clear association with enhanced STAT3 phosphorylation [81]. The elevation of GαS in liver cancer tissues underscores its critical function in HCC progression and prognosis, providing valuable insights for preventing hepatocarcinogenesis.

### Methylation and its implications in liver cancer

Emerging evidence indicates the crucial role of abnormal methylation patterns in the progression of liver cancer. The overexpression of protein arginine methyltransferases (PRMTs), which leads to abnormal methylation, is frequently associated with a poor prognosis in liver cancer [90, 91]. Recently, PRMT3 expression has been found to be significantly elevated in HCC, and this high expression is linked to poor clinical outcomes [92]. Moreover, elevated PRMT3 expression at both messenger ribonucleic acid (mRNA) and protein levels in oxaliplatin-resistant PLC-8024-R and Huh7-R cell lines pinpoints its pivotal role in oxaliplatin resistance. This positions PRMT3 overexpression as a potential biomarker for identifying oxaliplatin resistance in patients with liver cancer, linking higher PRMT3 levels to poorer outcomes and diminished therapeutic responses to oxaliplatin-based hepatic artery infusion chemotherapy (HAIC) [93]. Notably, elevated PRMT1 levels in HCC are linked to larger tumor size, increased microvascular invasion, elevated tumor, node, metastasis (TNM) stages, and poorer survival outcomes, positioning them as potential prognostic indicators [94]. As a key enzyme in serine biosynthesis, an increase in methylation levels of PHGDH and its enhanced enzyme activity in HCC tissues correlates with poor patient outcomes [95]. In addition, the significantly higher arginine methylation of heat shock protein A8 (HSPA8) in tumor tissues correlate with diminished overall survival rates among patients, underscoring their potential as prognostic indicators for liver cancer [96].

**Fig. 3:**
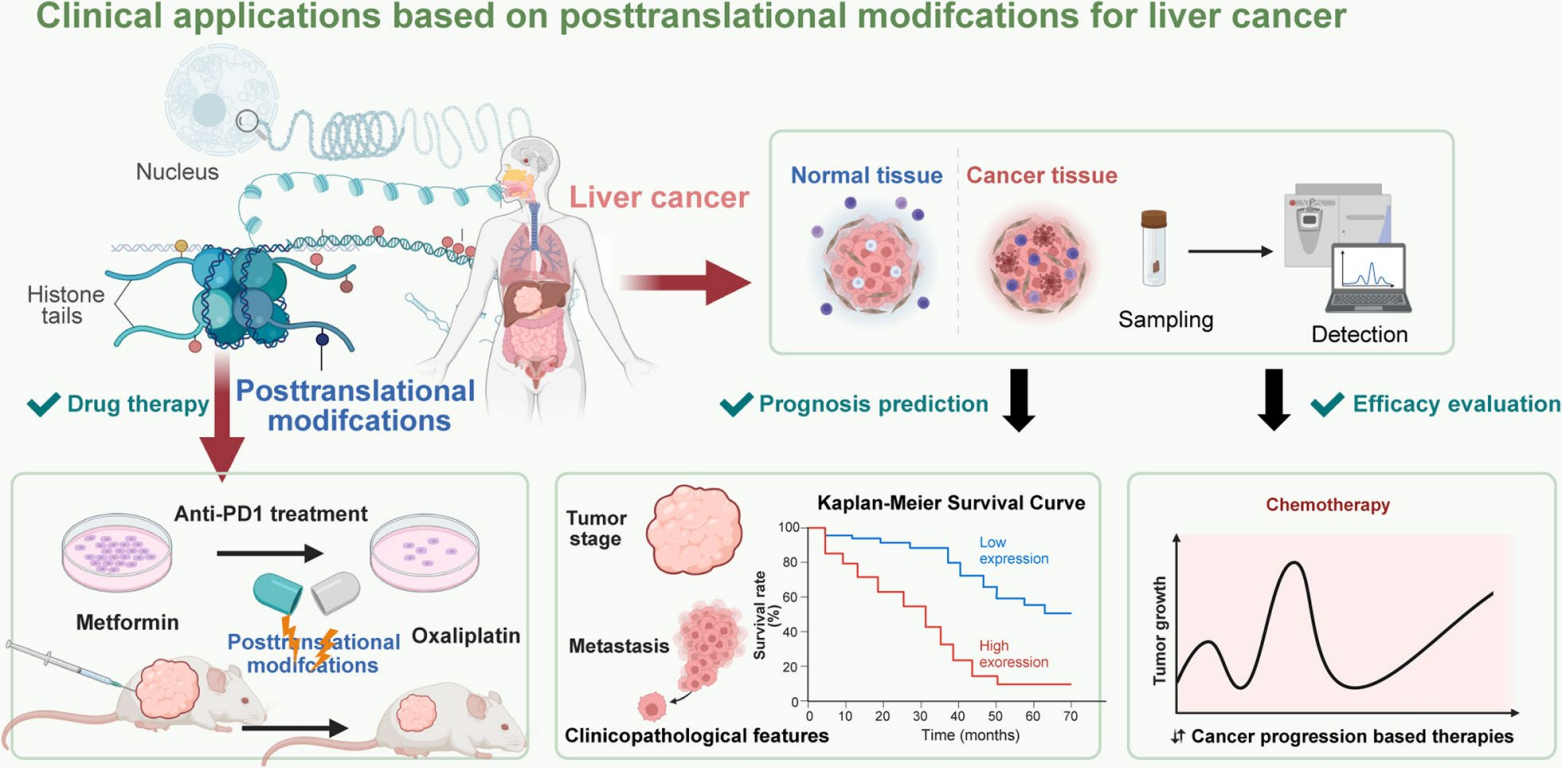
Clinical significance of multiple post-translational modifications in liver cancer management. The aberrant expression of PTMs, including phosphorylation, acetylation, methylation, and glycosylation, is linked to unfavorable clinical pathologies and prognosis in liver cancer, underlining their significance as prognostic biomarkers. Additionally, these PTMs have been identified as markers of resistance to chemotherapy, highlighting their utility in guiding treatment strategies. Given the complex involvement of PTMs in the pathogenesis of liver cancer, interventions targeting these modifications have demonstrated promising results in impeding tumor proliferation across various preclinical models. This insight reinforces the value of PTMs as potential therapeutic targets, paving the way for more effective liver cancer treatments

Emerging research has highlighted that methylation modifications orchestrate a wide array of cellular processes, contributing to the development and progression of HCC [91, 97–99]. Methylation processes play a pivotal role in the oncogenesis of HBV-induced HCC, specifically through the modulation of the hepatitis B virus X (HBx) protein [100–104]. Recent studies have illuminated the critical involvement of arginine methylation in regulating HBx-induced ferroptosis, a crucial process in cancer progression [102, 105–107]. HBx is found to elevate the expression of PRMT9 in HCC HepG2 and Huh7 cells, leading to increased arginine methylation of HSPA8. This modification significantly upregulates CD44 expression, contributing to the suppression of ferroptosis and fostering tumor growth and cell proliferation. This intricate understanding of HBx-induced HCC highlights the therapeutic potential of targeting arginine methylation pathways in HBV-related liver cancer management [96]. Recent research has highlighted that methylation also plays a significant role in regulating the resistance of liver cancer cells to conventional therapies [108]. PRMT3 mediates the methylation of insulin-like growth factor 2 mRNA-binding protein 1 (IGF2BP1), which in turn stabilizes the mRNA of heart development protein with EGF-like domains 1 (HEG1) in an m6A-dependent manner [93]. This process promotes the proliferation and survival of liver cancer cells, contributing to oxaliplatin resistance, confirmed through in vitro and in vivo experiments. These insights shed light on the intricate role of methylation in the adaptive mechanisms of liver cancer to chemotherapy, potentially guiding the stratification of patients for oxaliplatin-based HAIC therapy and the development of targeted interventions to overcome drug resistance. The association between methylation and metabolic deregulation has been found to affect the pathogenesis of HCC. The enzyme PRMT1 is identified as the mediator of phosphoglycerate dehydrogenase (PHGDH) methylation, which in turn elevates its catalytic efficiency. As a key enzyme in serine biosynthesis, an increase in methylation levels of PHGDH and its enhanced enzyme activity in HCC tissues correlates with poor patient outcomes. This augmentation of PHGDH activity boosts serine production, mitigates oxidative stress, and eventually promotes HCC cell proliferation and tumor growth. Notably, in the HCC patient-derived xenograft (PDX) and subcutaneous HCC cell-derived xenograft models, the use of a trans-activator of transcription (TAT)-tagged non-methylatable peptide to block PHGDH methylation effectively inhibits serine synthesis and suppresses HCC growth [95]. This finding highlights the potential of targeting PHGDH methylation as a novel therapeutic intervention for liver cancer by disrupting critical metabolic dependencies involved in tumor growth and survival. Moreover, recent research has highlighted the intricate interplay between methylation modifications and ubiquitination processes, revealing their combined impact on accelerating HCC progression [109]. PRMT5, by methylating the tumor suppressor retinoic acid receptor-related orphan receptor α (RORα), enhances its interaction with the E3 ubiquitin ligase itchy E3 ubiquitin protein ligase (ITCH). This interaction promotes the ubiquitination and subsequent degradation of RORα. Interestingly, this process is mitigated by oxidative stress-induced reactive oxygen species (ROS), which reduce PRMT5 protein levels, thereby restoring RORα expression. Elevated ROS levels, under specific oxidative stress conditions, are shown to inhibit the proliferation, migration, and invasion of HCC HepG2 cells [110]. Thymidine kinase 1 (TK1) has also been implicated in the metabolic reprogramming associated with HCC progression, orchestrating the complex interplay between methylation and ubiquitination. TK1 interacts with PRMT1, stabilizing it by inhibiting ubiquitination and subsequent degradation mediated by tripartite motif containing 48 (TRIM48). Increased PRMT1 levels drive the methylation of phosphofructokinase/fructose bisphosphatase type-3 (PFKFB3), leading to enhanced glycolysis, proliferation, migration, and invasion of tumor cells [94]. The involvement of methylation in altering cellular metabolism presents promising therapeutic opportunities, underscoring the potential to target methylation as a strategy for metabolic reprogramming in HCC treatment (Fig. 3).

### Glycosylation and its implications in liver cancer

Glycosylation profoundly influences HCC progression by altering a wide array of pro-tumorigenic molecules and signaling pathways across the disease’s various stages [59, 111]. It has also demonstrated that beta-1,3-galactosyltransferase 5 (B3GALT5) is overexpressed in HCC, correlating with poor prognostic outcomes [112]. Moreover, the upregulation of soluble carrier family 35 member A2 (SLC35A2) in HCC tissues, particularly those with lymph node infiltration or metastasis, underscores the enhancement of HCC’s metastatic potential [113]. The initiation of O-glycosylation by polypeptide N-acetylgalactosaminyltransferases (GALNTs) has been observed to reduce median survival in a mouse liver cancer model [114].

A key modulation involves the crosstalk between O-linked N-acetylglucosamine (O-GlcNAc) glycosylation, adenosine diphosphate (ADP)-ribosylation, and ubiquitination, crucially enhancing the DNA damage response during HCC progression. Enhanced O-GlcNAcylation of ADP-ribose glycohydrolase (PARG) mitigates the autoubiquitination of DNA damage-binding protein 1 (DDB1), thus stabilizing DDB1. The stabilization of DDB1 is critical for the targeted degradation of the oncogene cellular myelocytomatosis oncogene c-Myc in HCC Huh7 cells, thereby effectively impeding HCC tumorigenesis [115]. Concurrently, the impact of glycosylation extends beyond DNA repair to the metabolic landscape of HCC. Enhanced B3GALT5 activity facilitates the glycosylation of mTOR, activating its downstream effector, p70 ribosomal protein S6 kinase (p70s6k) [112]. This activation propels glycolysis, fostering cell proliferation and contributing to the development of HCC. This mechanistic understanding accentuates the pivotal role of glycosylation in manipulating cellular metabolism, driving the aggressive behavior of liver cancer cells [116, 117]. Consequently, targeting the glycosylation-mediated activation of the mTOR/p70s6k pathway presents a promising avenue for therapeutic intervention in HCC. The importance of glycosylation in HCC metastasis further exemplifies its role in cancer progression. Recent research underscores the critical influence of glycosylation modifications on the invasive phenotype associated with intrahepatic metastasis in liver cancer. Studies show increased activity of GALNTs, relocating from the Golgi apparatus to the endoplasmic reticulum (ER). This relocation facilitates the glycosylation of matrix metalloproteinase 14 (MMP14) by ER-targeted GALNT1 (ER-G1), markedly enhancing matrix degradation and tissue invasion, thereby promoting metastasis [114]. The altered expression of SLC35A2 recruits β-1,4-galactosyltransferase (B4GalT1) to the Golgi apparatus in HCC cells. The interaction between SLC35A2 and B4GalT1 in the Golgi apparatus drives the invasive capabilities of HCC cells, emphasizing the therapeutic potential of targeting these glycosylation pathways [113]. Additionally, β-galactoside α2,6 sialyltransferase 1(ST6GAL1) has been considered as a suppressor in HCC metastasis. By modulating the sialylation of melanoma cell adhesion molecule (MCAM), ST6GAL1 inhibits the migration and invasion of HCC cells, showcasing novel avenues through which glycosylation can influence cancer progression [118]. Collectively, these insights highlight the multifaceted role of glycosylation in HCC progression, from metabolic reprogramming to invasion and metastasis. Targeting glycosylation offers a promising therapeutic strategy to disrupt the molecular interplay driving HCC progression.

Given the significance of PTMs in liver cancer, our review highlights their potential as promising prognostic markers in liver cancer. We also elaborate on the regulatory mechanisms of PTMs in liver cancer pathogenesis, emphasizing the applications of PTMs as innovative alternatives to existing therapeutic strategies for liver cancer. Targeting specific PTMs precisely modulates cancer-related pathways and achieves promising results in diverse preclinical trials, suggest that further validation in large clinical cohorts is essential. Future clinical studies should evaluate the efficacy of these therapies across diverse patient populations, assess long-term safety and side effects. Comparative studies with existing treatments will help determine their overall therapeutic value, while biomarker development will aid in personalizing treatment plans. Moreover, incorporating green nanomaterials into PTM-targeted therapies presents a sustainable and effective approach to liver cancer treatment. Green nanomaterials, synthesized through eco-friendly processes, offer significant advantages with the minimized use of toxic solvents and enhanced biocompatibility in biomedical applications, including liver cancer therapy [119–124]. Advanced HCC often exhibits strong resistance to chemotherapy, with traditional drugs failing to achieve satisfactory therapeutic efficacy. Recent advances in nanotechnology, bioengineering, and chemical biology have led to new approaches to improve the efficacy and safety of liver cancer treatments [125–129]. In recent years, various nanoparticles and nanoparticle drug delivery systems have been extensively explored to enhance the therapeutic efficacy of the oral kinase inhibitor sorafenib in HCC [130]. Combining PTM-targeted modifications with nanomaterials represents a meaningful direction for future liver cancer therapies. This strategy holds promise for improving treatment outcomes and providing more effective therapeutic options for patients with liver cancer.

## Conclusion

The worse survival rates for advanced liver cancer underscore the critical need for a deeper understanding of the disease’s molecular underpinnings to bolster early detection and develop innovative treatment strategies. An in-depth investigation of epigenetic alterations in HCC progression has revealed the pivotal roles of various PTMs in maintaining normal physiological activities and their contribution to the pathogenesis and progression of diseases. Several studies identify PTMs as key drivers in the malignant progression of liver cancer, with a wide range of aberrant PTMs observable at different stages of the disease. These biochemical alterations, including phosphorylation, acetylation, methylation, and glycosylation, modify proteins at the post-translational level, impacting their functionality and downstream cancer-related signaling pathways. Therefore, abnormalities in multiple PTMs contribute to an array of malignant cellular activities, such as cell proliferation, migration, autophagy, chemoresistance, immune evasion, and various metabolic reprogramming. Furthermore, as studies delve deeper into the mechanisms of PTMs in the pathogenesis of liver cancer, targeted biomarkers and therapeutic approaches focusing on PTMs have shown promising clinical outcomes in preclinical experiments. Although various PTMs and their impacts on HCC pathogenic processes have been studied, there exist challenges in translating basic research insights on PTMs in liver cancer into clinical practice. The dynamic properties of PTMs and their context-dependent effects complicate delineating their precise contributions to the pathogenesis and progression of HCC. Most importantly, the ubiquity of PTMs in normal physiological processes and potential off-target effects significantly increase the difficulty in developing drugs targeting specific PTMs.

In summary, PTMs play a prominent role in driving the development and progression of liver cancer. Mounting evidence has revealed that dysregulated PTMs are implicated in the regulation of multiple biological processes by affecting the post-transcriptional modification of the associated genes. This promises PTMs to be more precise and effective therapeutic targets and prognostic markers to help improve the prognosis of liver cancer. Continued exploration of the molecular mechanisms into the intricacies of PTMs with HCC carcinogenesis is vital for unlocking new avenues for the treatment of liver cancer.

## Data Availability

Not applicable.

## Abbreviations

HCC: Hepatocellular carcinoma
PTMs: Post-translational modifications
HBV: Hepatitis B virus
HCV: Hepatitis C virus
NAFLD: Non-alcoholic fatty liver disease
CKB: Creatine kinase B
TCGA: The Cancer Genome Atlas
IHC: Immunohistochemistry
IGF1R: Insulin-like growth factor 1 receptor
GPX4: Glutathione peroxidase 4
LOXL3: Lysyl oxidase-like 3
EGF: Epidermal growth factor
DHODH: Dihydroorotate dehydrogenase
anti-PD1: Anti-programmed cell death 1
ZFP64: Zinc finger protein 64
PKCα: Protein Kinase C alpha
CSF1: Colony stimulating factor 1
TME: Tumor microenvironment
PCK1: Phosphoenolpyruvate carboxykinase 1
HSPA8: Heat shock protein A8
